# Silencing CK19 regulates ferroptosis by affecting the expression of GPX4 and ACSL4 in oral squamous cell carcinoma in vivo and in vitro

**DOI:** 10.1038/s41598-024-65079-0

**Published:** 2024-07-10

**Authors:** Yong Rao, Jingying Li, Lijuan Shi, Xiao Chen, Yun Hu, Yalin Mao, Xiaoyan Zhang, Xuqian Liu

**Affiliations:** 1 Department of Periodontics & Oral Mucosal Diseases, The Affiliated Stomatological Hospital, Southwest Medical University, Luzhou, 646000 Sichuan China; 2 Luzhou Key Laboratory of Oral & Maxillofacial Reconstruction and Regeneration, Luzhou, Sichuan China; 3 Department of Oral Medicine, Sichuan Vocational College of Traditional Chinese Medicine, Mianyang, 621000 Sichuan China; 4 Department of Orthodontics, Mianyang Stomatological Hospital, Mianyang, 621000 Sichuan China

**Keywords:** CK19, OSCC, Ferroptosis, GPX4, ACSL4, Biotechnology, Cancer, Molecular biology

## Abstract

To analyze the mechanism of how interfering with the cytokeratin 19 (CK19) pathway via the ferroptosis pathway affects tumor biological behaviors in the process of oral squamous cell carcinoma (OSCC) development. TCGA was used to analyze the expression of CK19 in pan-cancer and head and neck squamous cell carcinoma (HNSC) and to explore the ferroptosis-related genes related to HNSC. The effect of silencing CK19 on the migration ability of HSC-4 cells was verified by wound healing and migration assay. HSC-4 cells with silencing of CK19 and tumor-bearing nude mouse model were constructed. RT-qPCR, immunofluorescence and western blot were used to analyze the expression of ferroptosis-related genes. CK19 is highly expressed in human OSCC and nude mice. The migration ability of cells in the CK19-silenced group was lower than that of the control group. In vivo and in vitro, CK19 was negatively correlated with the expression of ACSL4 and positively correlated with the expression of GPX4. Compared with the control group, GPX4 expression was down-regulated and ACSL4 expression was up-regulated in the CK19-silenced group. Silencing CK19 also increased intracellular Fe^2+^ content and MDA content. Silencing CK19 can affect the expression of GPX4 and ACSL4 to regulate ferroptosis and at the same time increase the content of MDA, Fe^2+^ and ROS levels, thereby activating the regulation of ferroptosis pathway in the development of OSCC.

## Introduction

Exploring the precursor changes of oral mucosal epithelial cells in cancer progression and related prognostic biomarkers have become a hotspot for the early diagnosis and treatment of oral squamous cell carcinoma (OSCC), which aim to improve the diagnostic efficacy based on the specific diagnostic biomarkers in the pre-cancer stage or early stage of the cancerous lesions, and to intervene in the treatment as early as possible, so as to enhance the survival rate of the patients^1^. In the process of OSCC development, ferroptosis is promoted by induction of tumor cells, it can reduce the physiological effects of tumor resistance and inhibit tumor cell differentiation, invasion and metastasis^2^. Based on the above reasons, it has become the direction of our in-depth study of the development process of OSCC to explore the mechanism of tumor development process by exploring the pathway of targeted interference of tumor biomarkers associated with ferroptosis.

Cytokeratin is an important cytoskeletal protein that maintains the integrity of epithelial cells and can be used as a common tumor biomarker for OSCC diagnosis^3,4^. Among the cytokeratin subtypes, CK19, as a typical component of epithelial cells with a protein molecular weight of 40 kDa, is the smallest protein isolated from squamous cell carcinoma cells^5^. It mainly exists in monolayer epithelial cells and germinal layer cells, and is rarely found in normal oral mucosa and normal skin epidermis^6^. CK19 is important for predicting the metastatic potential of tumors such as thyroid, cervical and colon cancers and is a useful research tool for tumor diagnosis, treatment and prognosis^7^. Tanaka S et al. found that CK19 was involved in the invasion and metastasis of OSCC by comparing the clinicopathological features and survival of 100 squamous carcinoma patients, and CK19 may be a novel biomarker for OSCC^8^.

Previous studies have shown that CK19 can activate the Wnt/β-Catenin signaling pathway and participate in the development of epithelial ovarian cancer^9^. Wnt/β-Catenin signaling pathway can regulate the expression of glycogen synthase kinase-3 (GSK-3), and GSK-3 co-phosphorylates β-catenin with APC, Fzd and axin, resulting in β-catenin degradation through ubiquitin–proteasome pathway^10,11^. GSK-3 phosphorylates a set of Ser residues in the Neh6 domain of mouse NF-E2-related factor 2 (Nrf2) to promote its degradation, and Nrf2 is also regulated by GSK-3 activity^12–15^. Nrf2 serves as a crucial regulatory factor in ferroptosis, orchestrating the transcription of GPX4 and other pertinent genes to modulate iron homeostasis^16,17^. In addition, the expression of ACSL4 is also regulated by Nrf2, and some studies have found that TFR1 and ACSL4 can be down-regulated by activating the Nrf2 signaling pathway, thus upregulating GPX^18,19^. Therefore, we speculated that CK19 might be involved in the OSCC ferroptosis pathway by regulating ACSL4 and GPX4.

As a special programmed cell death regulation pathway, ferroptosis is driven by lipid ROS and regulated by iron ions^20^. It is significantly different from other types of death regulation mechanisms such as apoptosis, necrosis, autophagy and so on in cell morphology, biochemical and genetic characteristics^21^. GPX4 and ACLS4 have become the key factors in the regulation of ferroptosis pathway. Some studies have shown that both of them are related to the poor prognosis of OSCC^22^, but some contrary studies have shown that the expression of GPX4 may not be directly related to the prognosis of OSCC^23^. Studies have shown that down-regulation of GPX4 can inhibit tumor growth and affect disease progression in OSCC^24^. Studies have also shown that up-regulation of ACL4 transcription level or stable protein level can promote ferroptosis and inhibit tumor development^25^. With the in-depth study of the mechanism of ferroptosis, it has been found that promoting ferroptosis of cancer cells can inhibit cancer cell differentiation, migration and reduce drug resistance^21^. At present, there are few studies on CK19 and ferroptosis in OSCC, and its function and specific mechanism of action are not yet clear, which needs further study.

In this study, we intended to analyze the expression of CK19 and its effect on the prognosis of HNSC by TCGA, and re-confirm the differential expression of CK19 in the development of OSCC, and further explore the specific mechanism of how CK19 affects the ferroptosis pathway to participate in the regulation of OSCC development process through silencing CK19. At the same time, a nude mouse model of silencing CK19 was constructed to verify whether targeted silencing of CK19 affected the expression of ferroptosis-related genes such as GPX4 and ACSL4, thereby regulating ferroptosis of OSCC.

## Materials and methods

### Cell culture

Human OSCC cell lines (HSC4) were obtained from Sichuan University State Key Laboratory of Oral Diseases. Cells were cultured in DMEM culture medium (Thermo Fisher Scientific, China) with 1% cyan-stranded biclonal antibody and 10% fetal bovine serum (Solarbio, China) at 37 ℃ and 5% CO_2_. The solution was changed every 2 days. When cell fusion to 80%, cells were passaged for subsequent experiments.

### Experimental animals and specimens

Twelve SPF grade BALB/C male nude mice aged 3–4 weeks, weighing 15-18 g, were purchased from Beijing Huafukang Biotechnology Co., Ltd. (Certificate No.: SCXK (Beijing) 2019-0008) and raised in the Experimental Animal Center of Southwest Medical University. The animal study was reviewed and approved by all procedures were approved by the Southwest Medical University Animal Ethics Committee (Permit Number: 201905-7). All experiments were performed in accordance with relevant named guidelines and regulations. The study is reported in accordance with ARRIVE guidelines. All animals were anesthetized and sedated with sodium pentobarbital. Research investigators choose to euthanize mice by cervical dislocation.

A case of well differentiated squamous cell carcinoma surgically resected and pathologically diagnosed in the Affiliated Hospital of Southwest Medical University in March 2023 was collected. After fresh sampling, the specimens were fixed in 10% neutral formalin for 24 h, and then subjected to routine tissue treatment, paraffin embedding, and continuous 4 um tissue sections. The informed consent of the patient and her guardian has been obtained, and the informed consent has been signed. All experiments were performed in accordance with relevant named guidelines and regulations. The study was reviewed and approved by all procedures were approved by medical Ethics Committee of Affiliated Stomatological Hospital of Southwest Medical University (Permit Number: 20180510001).

### TCGA dataset analysis

The correlation between tumor and ferroptosis gene, the correlation between CK19 and cancer or normal tissues, and the expression of CK19 in various types of cancer were obtained from the TCGA dataset. KM survival analysis was performed using v4.0.3 version R software (R foundation for statistical computing, 2020) and survival status maps of head and neck cancer patients and CK19 were drawn. Spearman correlation analysis between CK19 and ferroptosis-related genes was performed using the R software package ggstatsplot.

### Screen the optimal infection MOI value and lentivirus

The construction of pHS-ASR-1115 (H5), pHS-ASR-1116 (H6) and pHS-ASR-1117 (H7) silencing lentivirus and pHS-ASR-LW429 (H9) empty lentivirus were completed by Shanghai Synbiotics. Five groups of H5, H6, H7, H9 and HSC-4 (blank control cells without reagents) were designed. Each group added different lentiviruses based on MOI = 40. After 24 h of transfection, observe the cell status and exchange fluids, extract total RNA from each group of cells, and perform RT-qPCR to measure the expression of CKl9 mRNA in each group. Interference sequence of lentivirus was shown in Supplementary Table 1.

### Construct silencing CK19 lentivirus and transfect HSC-4

CK19-shRNA fragment and silencing CK19 lentivirus was provided by Beijing Hesheng Gene Technology Co., Ltd. CK19-shRNA01, 5′-CCAGCGGCTCATGGACATCAA-3′; CK19-shRNA02, 5′-GCGAAGCCAATATGAGGTCAT-3′; CK19-shRNA03, 5′-CGAGAAGCTAACCATGCAGAA-3′. The shRNA DNA sequence was inserted into the lentiviral vector pLV-hU6-shRNA-Puro. Sequencing primer: pHS-ASR-1115, 5′-CGGGTTTATTACAGGGACAGCAG-3′; pHS-ASR-1116, 5′-CGGGTTTATTACAGGGACAGCAG-3′; pHS-ASR-1117, 5′-CGGGTTTATTACAGGGACAGCAG-3′. Lentivirus transfection: 24 h before transfection, HSC4 cells were digested and inoculated in 6-well plates. When the cell fusion rate reached 30%, the lentivirus was transfected into HSC4 cells, and the medium was changed after 12 h of culture, and the culture was continued. The cells were divided into silencing group, empty group and untransfected group.

### Real-time q-PCR analysis

Total RNA was extracted from each group of cells, the RNA concentration was determined, and the cDNA was reversely transcribed, and the SYBR method was used for the experiment. The relative expression of mRNA in each group was calculated using the 2^−ΔΔCt^ method. All primers were synthesized by Shanghai Shenggong Co., Ltd. The primer sequence was shown in Supplementary Table 2.

### Immunohistochemical analysis

The cells in the silencing group and the control group were prepared, and the cells were required to be evenly distributed on the slides, and 50–60% was appropriate. Rabbit anti-human ACSL4 polyclonal antibody (Abways, China) and rabbit anti-human GPX4 polyclonal antibody (Abways, China) were prepared according to 1:100, and PBS buffer was used as negative control. The slides were fixed with 10% neutral formaldehyde solution for 20 min and incubated with 3% hydrogen peroxide for 10 min. The primary antibody was incubated overnight at 4 ℃ and then incubated with goat anti-rabbit TRITC (Bioss, China) in the dark. The nuclei were stained with DAPI. Finally, the slide was sealed with glycerin, the fluorescence was observed, and the image was analyzed.

### Wound healing assay

The control group and the silencing group were prepared with 6 mL of cell suspension at a concentration of about 3–5 × 104 cells/mL, and 2 mL was added to the hole to ensure that the cell suspension was evenly spread on the bottom of the plate. Each group was repeated three times. On the second day, cells were cultured to be more than 90% confluence, the monolayer was scratched the tip of a 100 μL sterile pipette. After washing the scratched cells with PBS (Solarbio, China), serum-free medium was added. After 24 h and 48 h of cell culture in the silence group and the control group, the photographs were observed and recorded under the microscope (OLYMPUS, Japan).

### Cell migration assay

0.6 mL of 20% serum medium was added to the lower chamber of the cell chambers, and the upper chamber was inoculated with 0.1 mL of pure DMEM medium re-suspended at a concentration of 3 × 10^4^ cells/mL in the silencing and control groups. After 48 h of incubation, the cells at the bottom of the upper chamber were wiped off. The cell structure was fixed with 4% paraformaldehyde. 700 μL of 1:1000 DAPI solution (Solarbio, China) was added to each well of a 24-well plate and stained with light for 30 min. The DAPI solution was washed off. Five fields of view were randomly selected for counting under a Leica fluorescence microscope (OLYMPUS, Japan), and cells were counted by Image software.

### Western blot assay

Cells were lysed with RIPA tissue/cell lysate: PMSF (100:1, Solarbio, China). Protein concentration was determined using a BCA kit (Solarbio, China) at a volume ratio of A:B = 1:50. The samples were electrophoresed and transferred to PVDF membranes. The PVDF membrane was closed in skimmed milk for 1 h, incubated with primary antibody at 4℃ overnight and secondary antibody at room temperature for 1 h.

### MDA content assay

MDA levels were detected using an MDA detection kit (Solarbio, China) according to the instructions. MDA content (nmol/10^4^ cell) = (12.9 × (ΔA532–ΔA600)–2.58 × ΔA450) × V_total_ ÷ (400 ÷ V_extract_ × V_sample_) = 0.125 × (12.9 × (ΔA532–ΔA600)–2.58 × ΔA450).

### Cellular Fe^2+^ content assay

Fe^2+^ levels were measured using the Cellular Ferrous Ion Colourimetric Assay Kit (Elabscience, China) according to the instructions.

### Reactive oxygen (ROS) level assay

The cells in the silence group and the control group were inoculated on the plate, and each group was set up with 3 vice holes. After 24 h, the cells in the 6-well plate were observed. When the plate reached 85%, 2 mL of 10 μmol/L DHE probe was added, and 3 μL of 3% H_2_O_2_ was added to each well. The cells were cultured in the incubator away from light for 30 min. After incubation, the cells were washed three times with PBS and observed under a laser microscope (OLYMPUS, Japan).

### Subcutaneous tumor formation in nude mice

The two kinds of cells transfected with CK19 silencing lentivirus and empty lentivirus in good growth state were digested and resuspended in serum-free medium, 0.2 ml/only (final concentration of 1 × 10^7^) was inoculated subcutaneously in the right upper limb of nude mice according to the group to establish the animal model of tumor-bearing nude mice.

### HE staining

Nude mice with loaded tumors were sacrificed and then the transplanted tumors were embedded in conventional paraffin, and the tissue specimens were cut into 4 μm-thick sections. In a fume hood it was first dewaxed then soaked in hematoxylin stain, then fixed in xylene solution, and finally closed with an appropriate amount of neutral gum and observed under a microscope.

### TUNEL assay for detection of apoptosis

The stripped transplanted tumor tissue was fixed, embedded in paraffin, and sliced at 4 μm thickness, and then operated according to the instructions of the TUNEL detection kit. The results were immediately photographed under a fluorescence microscope and analyzed by Image J.

### Statistical analysis

SPSS25.0 and Graph Pad Prism were used for statistical processing and analysis. All quantitative results were expressed as mean ± standard deviation (Mean ± SD). When continuous variables met normal distribution and variance chi-square test showed that the variance was uniform, the methods used were independent samples *t*-test or ANOVA. For continuous variables that did not meet the normal distribution, the rank-sum test was used to perform the statistical analysis. For the categorical variables expressed as frequencies (percentages) and analyzed by the chi-square test. P < 0.05 was as statistical significance.

### Ethical approval

The animal study was reviewed and approved by all procedures were approved by the Southwest Medical University Animal Ethics Committee (Permit Number: 201905-7). The study was reviewed and approved by all procedures were approved by medical Ethics Committee of Affiliated Stomatological Hospital of Southwest Medical University (Permit Number: 20180510001).

## Results

### The expression of CK19 may not be related to the survival prognosis of patients with HNSC

**Figure 1:**
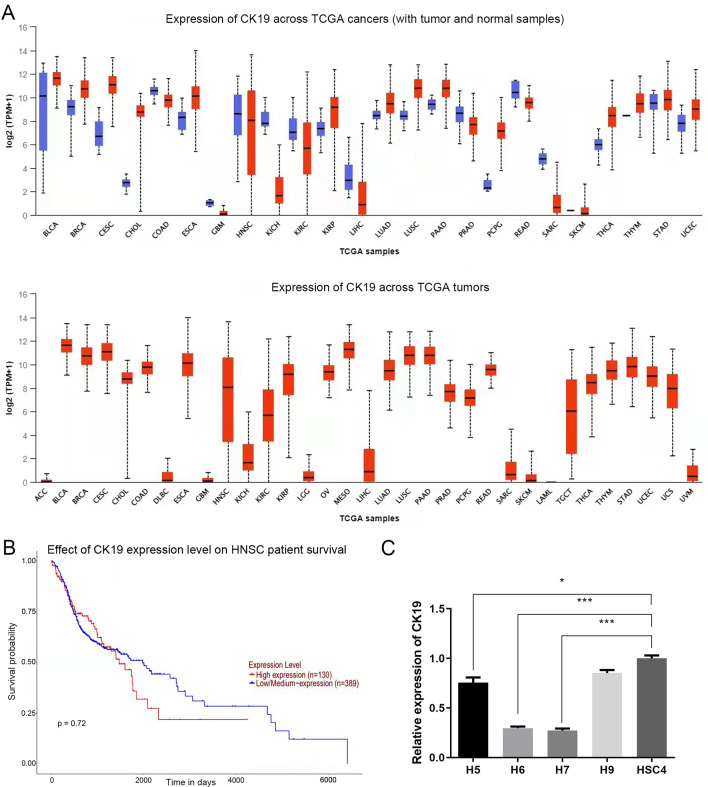
Results of the TCGA dataset analysis and RT-qPCR (**A**) Picture of expression of CK19 gene in tumor and normal tissue (**B**) KM survival curve distribution of survival time and survival status in patients with CK19 and HNSC tumors note: Distribution of CK19 and KM survival curves in the HNSC database of TCGA (p = 0.72), with a sample size of 130 for the high CK19 expression group and 389 for the low to moderate CK19 expression group. (**C**) The expression of CK19 in H5, H6, H7, H9 and HSC-4 untransfected groups. *P < 0.05, ***P < 0.001.

The TCGA dataset showed CK19 expression was not significantly different in HNSC, it was significantly down-regulated in KICH, LGG, ACC, DLBC, GBM, LIHC, SKCM, UVM, and SARC, and up-regulated in BLCA, BRCA, CESC, CHOL, COAD, ESCA, OV, and MESO (Fig. 1A). Figure 1B shows the relationship between CK19 expression level and survival prognosis of patients with HNSC, and ROX analysis suggested that the expression level of CK19 had no significant correlation with survival time of patients with HNSC.

### HSC-4 human oral squamous cell line was used as the experimental cell model

Due to the HSC-4 cell line had a high content of CK19, it was selected for subsequent experiments. When MOI = 40, the amount of virus used was the least, so the amount of virus with MOI = 40 was selected for subsequent experiments. As qPCR results showed, compared with other groups, the effect of lentivirus H6 silencing CK19 gene in HSC-4 cells was good (Fig. 1C). HSC-4 cells transfected with lentivirus pHS-ASR-1116 (H6) and empty lentivirus pHS-ASR-LW429 were respectively used as the silencing group and control group for subsequent experiments.

### Silencing CK19 could reduce the migration rate of HSC-4 cells

**Figure 2:**
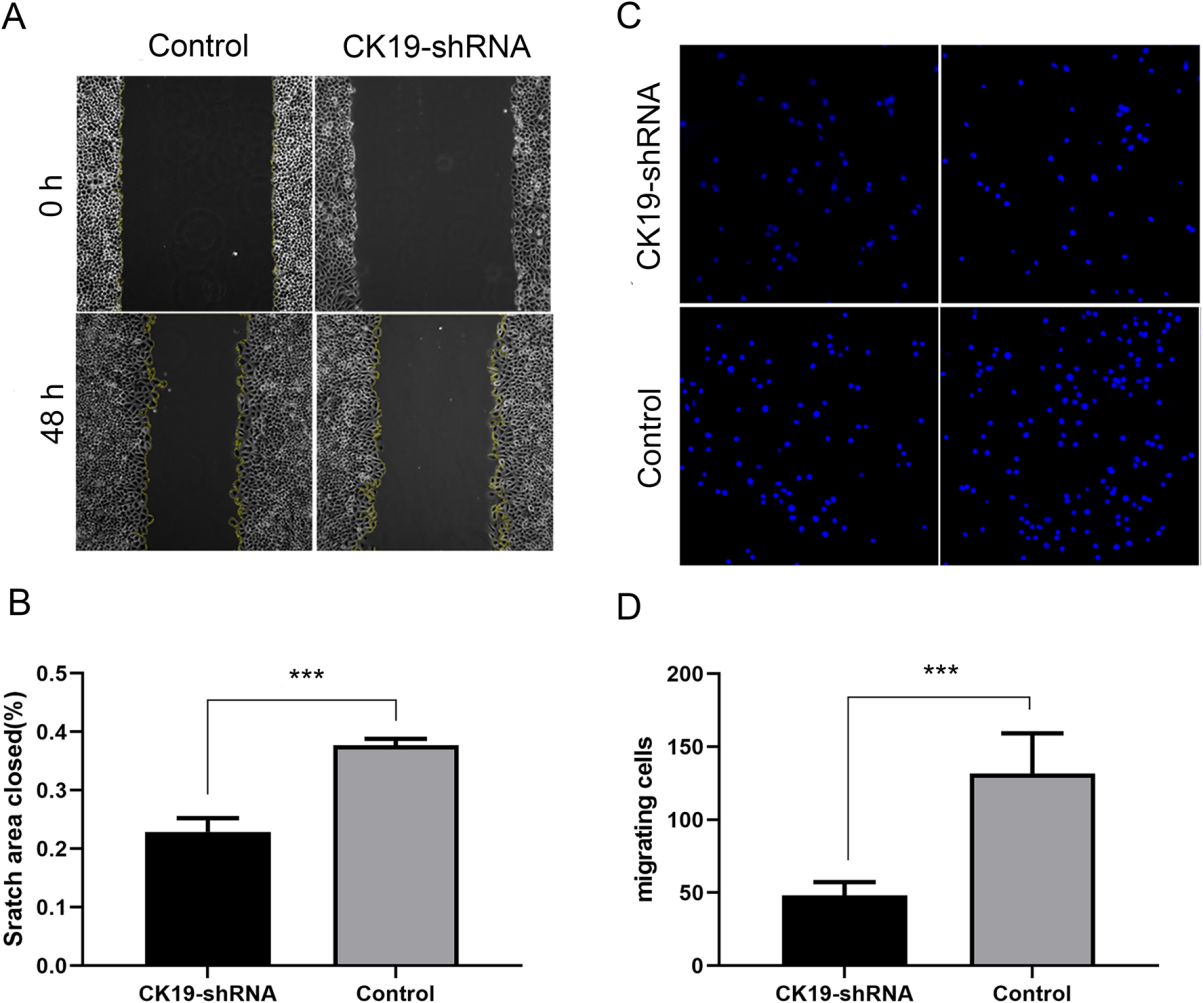
Wound healing assay and cell migration assay. (**A**) Observation of cell migration under the microscope, (**B**) Histogram of cell migration and healing area percentage, (**C**) Number of migrating cells observed under the microscope, (**D**) Histogram of the number of migrating cells. Date are shown as the mean ± SD (n ≥ 3). ***P < 0.001.

After 48 h of cell scratch test, the migration rate of the silencing group was lower than that of the control group. After silencing CK19, the migration rate of HSC-4 cells slowed down (Fig. 2A,B). After 24 h of cell migration in the silencing group and the control group, the number of migrated cells in the silencing group was lower than that in the control group. After silencing CK19, the migration rate of HSC-4 cells slowed down (Fig. 2C,D).

### ACSL4 and GPX4 acted as key genes for ferroptosis associated with CK19

**Figure 3:**
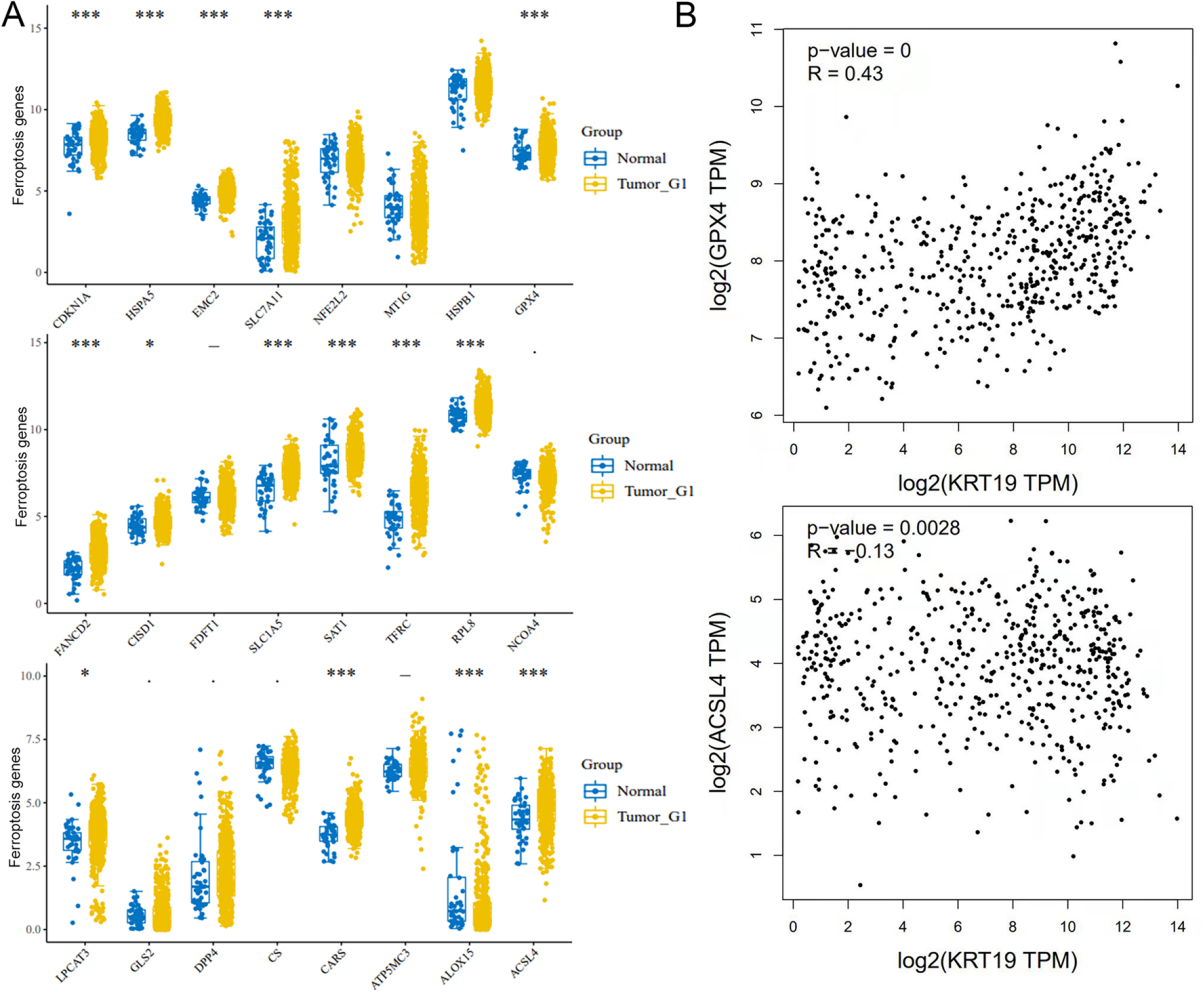
The relationship between ferroptosis-related genes and CK19 was analyzed by TCGA database. (**A**) Scatter plots of ferroptosis related genes associated with tumor and normal tissue (**B**) Correlation regression between CK19 gene and ferroptosis related genes ACSL4 and GPX4.

The TCGA database found that ferroptosis-related genes GPX4, ACSL4, CDKN1A, HSPA5, EMC2, SLC7A11, LPCAT3, CARS, ALOX15, FANCD2, CICD1, SLC1A5, SAT1, TFRC, and RPL8 were significantly different from normal tissues in various tumors (Fig. 3A). The linear regression analysis showed that ACSL4 was negatively correlated with CK19, while GPX4 was positively correlated with CK19. Therefore, GPX4 and ACSL4 were screened to represent the genes of ferroptosis as subsequent study (Fig. 3B).

### Silencing CK19 significantly increased the expression of ACSL4 and decreased the expression of GPX4

**Figure 4:**
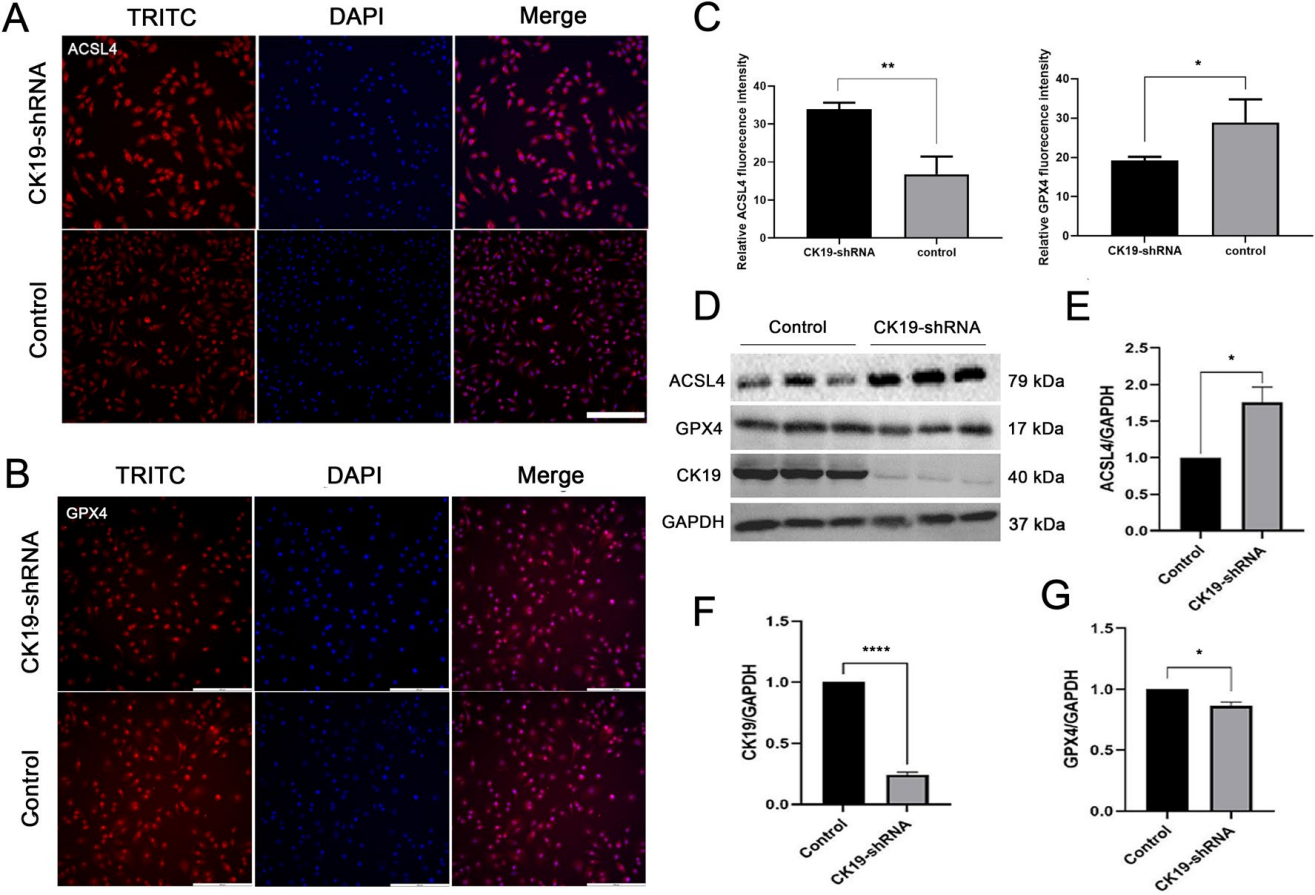
The expression of ACSL4 and GPX4 in silent and control groups. (**A**) Cellular immunofluorescence of ACSL4 (inverted microscope,  × 200) bar = 200 µm, (**B**) Cellular immunofluorescence of GPX4 (inverted microscope,  × 200) bar = 200 µm, (**C**) Fluorescence intensity statistics (**D**) Western blot analysis of CK19, ACSL4 and GPX4 (Original blots/gels are presented in Supplementary Fig. 1) (**E–G**) Gray value statistics. *P < 0.05, **P < 0.01.

The expression of ACSL4 and GPX4 in HSC-4 cells transfected with silencing CK19 and control groups were analyzed by immunofluorescence staining, and the results showed that ACSL4 and GPX4 were expressed in all groups, and mainly in the cytoplasm (Fig. 4A,B). By counting the fluorescence intensity, it was found that the expression of GPX4 was significantly lower and the expression of ACSL4 was significantly higher in the CK19-shRNA group, compared with control group (Fig. 4C). Furthermore, Western blot showed the similarly results as above (Fig. 4D–G).

### Silencing CK19 could increase content of MDA, ROS and Fe^2+^

**Figure 5:**
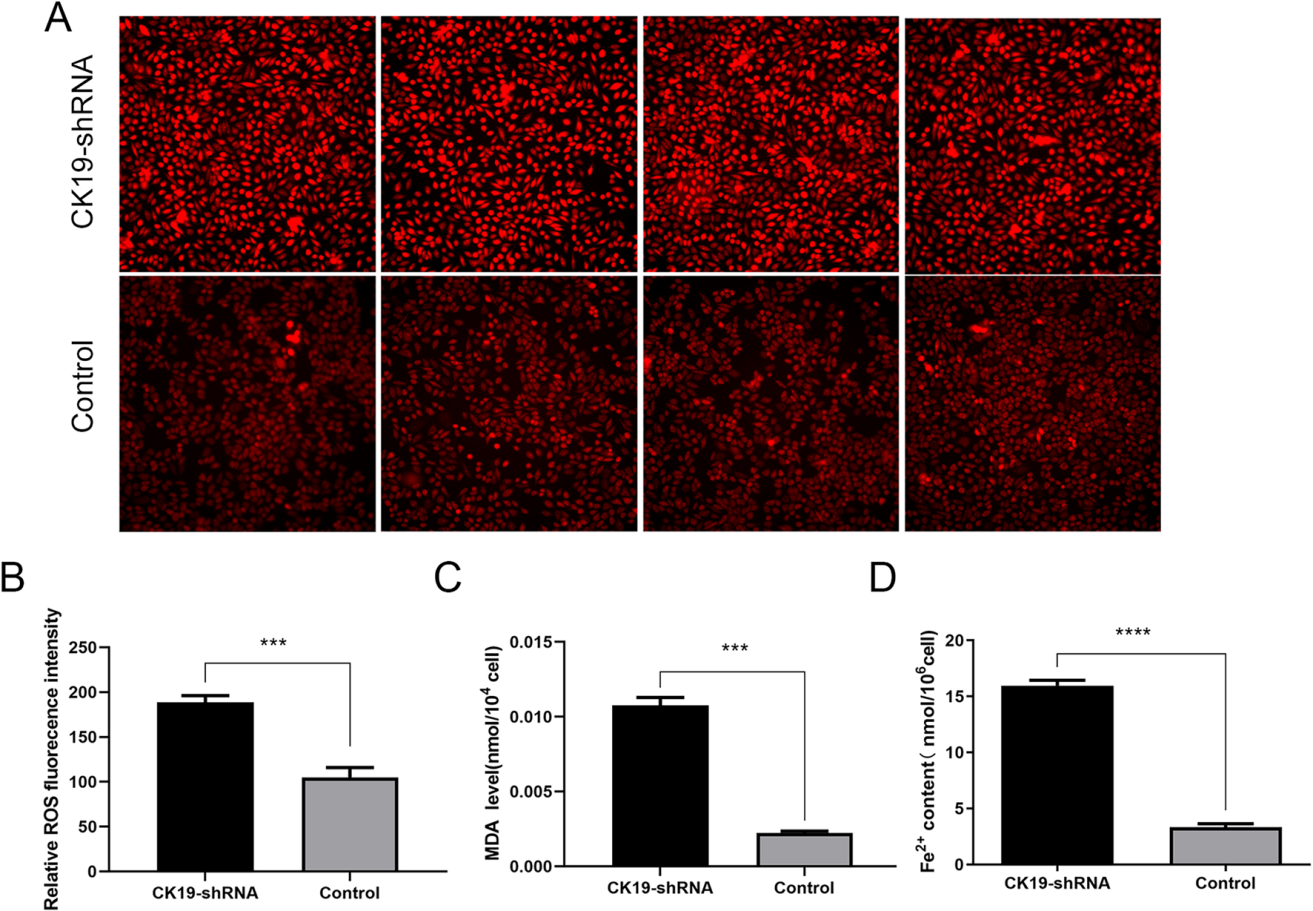
The content of MDA, ROS and Fe^2+^ in silent and control groups. (**A**) ROS fluorescence in silent and control groups. (× 200) bar = 200 µm (**B**) Fluorescence intensity statistics. (**C**) The content of MDA in silent and control groups. (**D**) The content of Fe^2+^ in silent and control groups. ***P < 0.001.

We measured the ROS, MDA and Fe^2+^ contents in the CK19-shRNA group and the control group respectively. The results showed that the content of ROS and MDA in the CK19-shRNA group was higher than that in the control group (Fig. 5A–C), indicating that the oxidative stress response of ferroptosis in HSC-4 cells was enhanced after CK19 silencing. And there were higher Fe^2+^ contents in the CK19-shRNA group (Fig. 5D).

### Silencing CK19 significantly increased the expression of ACSL4 and decreased the expression of GPX4 and could increase content of MDA and Fe^2+^ in nude mice

**Figure 6:**
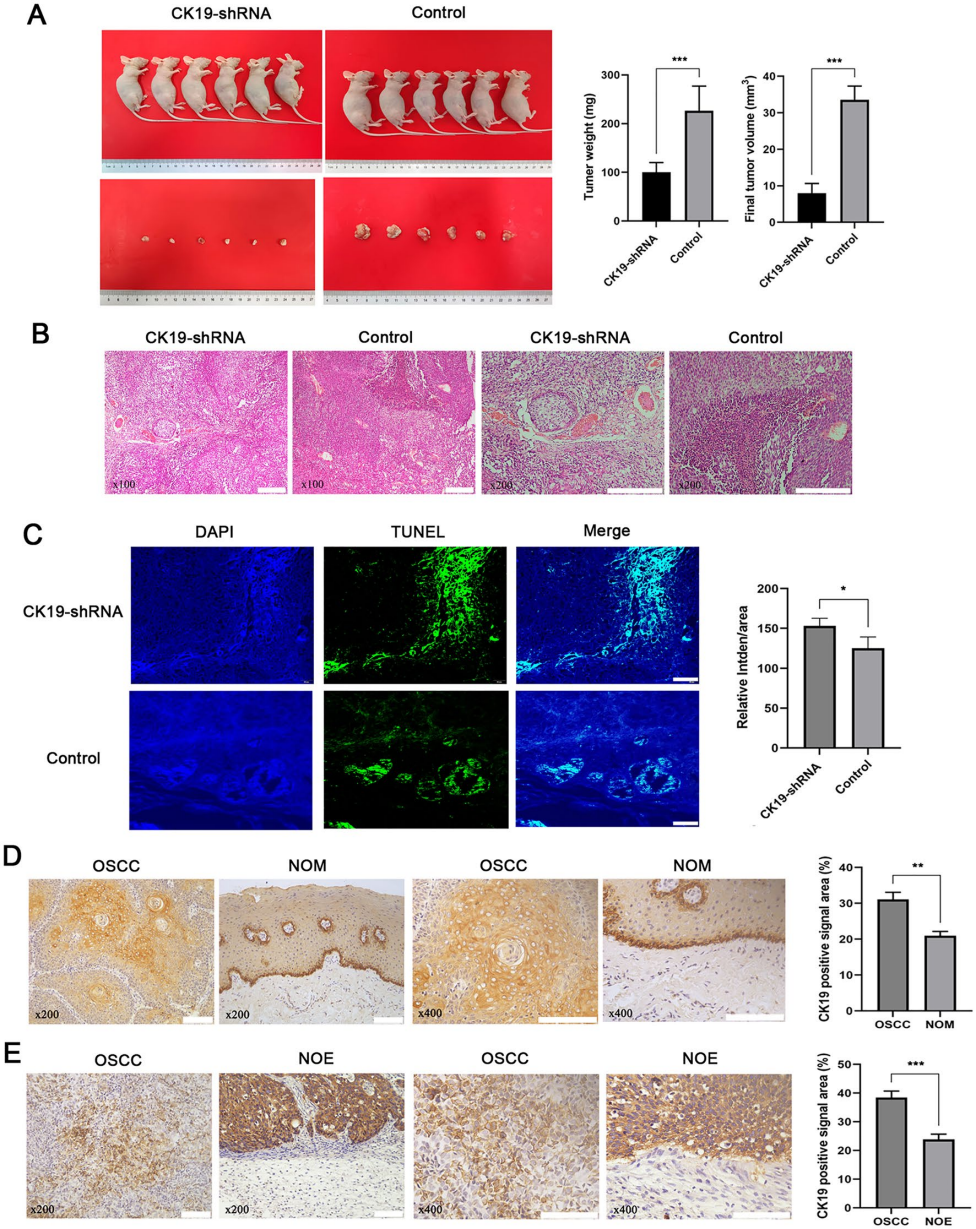
HE staining, TUNEL staining and immunohistochemical analysis. (**A**) Control and silent groups stripped of transplanted tumors and statistical analysis of weight volume. ***P < 0.001 (**B**) HE staining of stripped graft tumor tissue in both groups. Bar = 200 µm (**C**) TUNEL staining of tumor tissue (× 200) and average fluorescence intensity statistics. Bar = 100 µm, *P < 0.05 (**D**) The expression of CK19 in oral squamous cell carcinoma and normal oral mucosa and statistical analysis. Bar = 100 µm, **P < 0.01 (**E**) The expression of CK19 in tumor-bearing nude mice and normal epithelium of nude mice and statistical analysis. Bar = 100 µm, ***P < 0.001.

We inoculated nude mice with CK19-shRNA group and control group cells, and found that 12 nude mice all formed tumors after 30 days, and the tumor formation rate was 100%. In the CK19-silenced group, the tumor volume and weight were significantly smaller than that of the control group (Fig. 6A). The tumor tissues of the two groups of nude mice had heterogeneity and obvious tumor nodule structure, the cells were irregularly arranged, and the nuclear-to-cytoplasmic ratio of the cells increased, and the nuclear size was different, showing the characteristics of tumor cells. The control group could see obvious tumor necrosis area (Fig. 6B). Functional experiments were performed to determine the role of silencing CK19 in OSCC tumor cells in vivo. TUNEL staining results showed that silencing CK19 could promote apoptosis of tumor cells in vivo (Fig. 6C). Immunohistochemical staining results showed that the expression of CK19 in OSCC tissue was significantly higher than that in normal oral mucosa (Fig. 6D), and the expression of CK19 in tumor-bearing nude mice was significantly higher than that in normal epithelium of nude mice (Fig. 6E).

**Figure 7:**
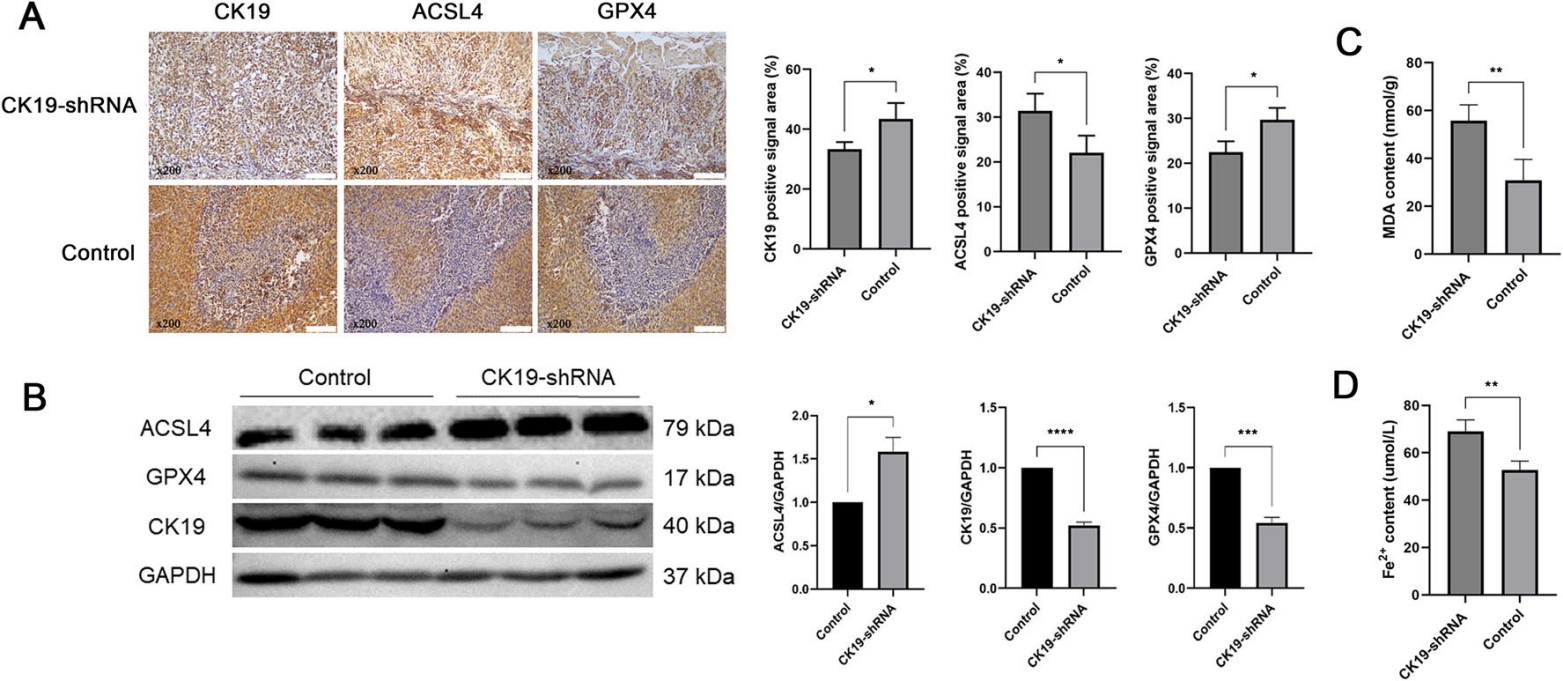
The expression of ACSL4 and GPX4 in silent and control groups by immunohistochemical analysis and western blotting. (**A**) The expression of ACSL4, GPX4 and CK19 in silent and control groups and statistical analysis bar = 100 µm, *P < 0.05 (**B**) Western blot showed the relative expression of CK19, ACSL4 and GPX4 in silent and control groups and statistical analysis (Original blots/gels are presented in Supplementary Fig. 1). *P < 0.05, **P < 0.01 (**C**,**D**) The MDA and Fe^2+^ content in transplanted tumors. **P < 0.01.

Further, ACSL4 was highly expressed and GPX4 was lower expressed in transplanted tumors in CK19-shRNA mice group (Fig. 7A). Western blot was used to analyze the expression of each protein in CK19 silencing group and control group. CK19, GPX4 protein levels were significantly lower and ACSL4 levels were significantly higher in the CK19-silenced group compared to the control group (Fig. 7B). Detection of Fe^2+^ levels and MDA content within stripped graft tumor serum showed that silencing CK19 significantly increased Fe^2+^ levels and MDA content (Fig. 7C,D).

## Discussion

In this study, the mRNA expression profile of CK19 and the clinical information of HNSC patients were collected from the TCGA database for bioinformatics analysis. We found that CK19 was significantly expressed in pan-cancer tissues, and there was no significant difference in the expression of CK19 between tumor and normal tissues. This is contrary to the conclusion that CK19 is significantly highly expressed in tumor tissues found in previous studies and clinical practice^26,27^, which needs further experimental verification. Through univariate Cox analysis and KM analysis, the prognostic gene characteristic curve was established. It was found that there was no significant statistical difference in CK19 gene expression difference analysis and survival analysis of HNSC, which may not be directly related to prognosis. However, this experiment showed that the migration ability of HSC4 cells was weakened after silencing CK19 through cell scratch test and migration test. It was also verified by immunohistochemistry that the expression of CK19 in human squamous cell carcinoma tissues and mouse tumor-bearing tissues was higher than that in normal tissues. This result was consistent with the previous study that the expression level of CK19 in the development process of OSCC was up-regulated with the degree of abnormal hyperplasia of oral mucosal epithelium^28–30^. The above shows that the data of HNSC as a sample in the bioinformatics analysis cannot objectively reflect the expression trend of CK19 in OSCC and normal oral mucosa, which may be because OSCC is only a part of HNSC.

The effect of silencing CK19 on tumor growth and apoptosis was further investigated from the perspective of in vivo experiments by establishing a nude mouse xenograft model. The results showed that the final weight and volume of transplanted tumors in the silent CK19 group were significantly lower than those in the control group, and the apoptotic cells in the tumor tissues of the silent CK19 group were significantly increased. These results indicate that after silencing CK19, the tumor grows slowly, the tumor cells are well differentiated, the degree of malignancy is reduced, and the apoptosis of tumor cells can be promoted. As in a study, the release of serum CYFRA 21-1 (a fragment of CK19) in liver cancer cells is closely related to tumor cell apoptosis^31^.

Ferroptosis is a novel form of programmed cell death that is different from apoptosis, necrosis or autophagy, and is an anti-tumor mechanism that is essential for eliminating malignant cells^32^. Hassannia B et al. found that ferroptosis-related genes FRGs and pathways may also be involved in the development of OSCC and can be used to develop prognostic markers^33^. Tumor cells have a greater demand for iron than normal cells, which makes them more susceptible to ferroptosis^33^. When iron overload occurs in cells, excessive free Fe^2+^ reacts with H_2_O_2_ to produce a large number of toxic products and ROS to promote ferroptosis^34^. Ferroptosis is controlled by GPX4, and GPX4 inactivation is thought to lead to cell death^35^. In this study, TCGA database was used to analyze the differences of ferroptosis-related genes between oral cancer and adjacent normal tissues, and a variety of differentially expressed ferroptosis genes were screened out. The correlation analysis between CK19 and candidate differential genes showed that two classical ferroptosis-related genes ASCL4 and GPX4 were significantly correlated with CK19. And studies had shown that CK19 could enhance the invasion, migration and proliferation of cancer cells through the Wnt/β-catenin pathway^9^. CK19 may regulate the expression of GSK-3 by activating Wnt/β-Catenin signaling pathway, and then affect AMPK/Nrf2/GPx4 pathway, and finally regulate the expression of GPX4 and ACSL4^11–13,16^. GPX4 can convert lipid hydroperoxides into fatty alcohols. Inhibition of GPX4 expression leads to lipid peroxidation and can lead to ferroptosis^25,36,37^. Studies have shown that ionizing radiation can induce the expression of ACSL4, leading to lipid peroxidation and ferroptosis^38–40^. This is consistent with our findings that silencing CK19 promotes ferroptosis and inhibits tumor growth by activating ACSL4 expression and inhibiting GPX4 expression. However, the pathways through which CK19 completes this process are still unclear, and further research is needed.

Ferroptosis can cause dysfunction of the antioxidant system and lead to a decrease in the overall antioxidant capacity of cells^41^. Mitochondria are damaged with the increase of external stimuli. When mitochondria are severely damaged, they lose the ability to regulate ROS, which further leads to an increase in ROS^41^. Ferroptosis is also closely related to the content of MDA and Fe^2+^. Previous studies have also used the content of MDA and Fe^2+^ as key indicators to explore the regulation of ferroptosis in OSCC^42–44^. We found that the content of Fe^2+^ and MDA in the silent CK19 group was significantly higher than that in the control group, indicating that silencing CK19 can promote ferroptosis. In the future, we will verify our conclusions by restoring CK19, overexpressing CK19, overexpressing ACSL4 and GPX4 and using other ferroptosis inhibitor and activator.

In summary, silencing CK19 can affect the expression of GPX4 and ACSL4 to regulate ferroptosis and at the same time increase the content of MDA, Fe^2+^ and ROS levels, thereby activating the regulation of ferroptosis pathway in the development of OSCC.

### Supplementary Information

Supplementary Figure 1.

Supplementary Tables.

## Data Availability

The datasets generated during and/or analyzed during the current study are not publicly available, but are available from the corresponding author on reasonable request.

## References

[1] Liu J (2021). circIGHG-induced epithelial-to-mesenchymal transition promotes oral squamous cell carcinoma progression via miR-142-5p/IGF2BP3 signaling. Cancer Res..

[2] Lei G, Zhuang L, Gan B (2022). Targeting ferroptosis as a vulnerability in cancer. Nat. Rev. Cancer.

[3] Kuburich NA, den Hollander P, Pietz JT, Mani SA (2022). Vimentin and cytokeratin: Good alone, bad together. Semin. Cancer Biol..

[4] Vaidya M, Dmello C, Mogre S (2022). Utility of keratins as biomarkers for human oral precancer and cancer. Life (Basel).

[5] Lu Q (2020). CK19 promotes ovarian cancer development by impacting on Wnt/β-catenin pathway. OncoTargets Ther..

[6] Yoshida K (2015). Expression of cytokeratin 14 and 19 in process of oral carcinogenesis. Bull. Tokyo Dent. Coll..

[7] Mehrpouya M, Pourhashem Z, Yardehnavi N, Oladnabi M (2019). Evaluation of cytokeratin 19 as a prognostic tumoral and metastatic marker with focus on improved detection methods. J. Cell. Physiol..

[8] Tanaka S (2020). Cytokeratin 19 as a biomarker of highly invasive oral squamous cell carcinoma with metastatic potential. J. Oral Maxillofac. Surg. Med. Pathol..

[9] Lu Q, Qu H, Lou T, Liu C, Zhang Z (2020). CK19 promotes ovarian cancer development by impacting on Wnt/β-catenin pathway. OncoTargets Ther..

[10] Janda CY, Waghray D, Levin AM, Thomas C, Garcia KC (2012). Structural basis of Wnt recognition by frizzled. Science.

[11] Marchetti B (2018). Wnt/β-catenin signaling pathway governs a full program for dopaminergic neuron survival neurorescue and regeneration in the mptp mouse model of Parkinson’s disease. Int. J. Mol. Sci..

[12] Rada P (2011). SCF/{beta}-TrCP promotes glycogen synthase kinase 3-dependent degradation of the Nrf2 transcription factor in a Keap1-independent manner. Mol. Cell. Biol..

[13] Chowdhry S (2013). Nrf2 is controlled by two distinct β-TrCP recognition motifs in its Neh6 domain, one of which can be modulated by GSK-3 activity. Oncogene.

[14] Hayes JD, Chowdhry S, Dinkova-Kostova AT, Sutherland C (2015). Dual regulation of transcription factor Nrf2 by Keap1 and by the combined actions of β-TrCP and GSK-3. Biochem. Soc. Trans..

[15] Cuadrado A (2015). Structural and functional characterization of Nrf2 degradation by glycogen synthase kinase 3/β-TrCP. Free Radic. Biol. Med..

[16] Kerins MJ, Ooi A (2018). The roles of NRF2 in modulating cellular iron homeostasis. Antioxid. Redox Signal..

[17] Staerck C (2017). Microbial antioxidant defense enzymes. Microb. Pathog..

[18] Li XN (2024). Caffeic acid alleviates cerebral ischemic injury in rats by resisting ferroptosis via Nrf2 signaling pathway. Acta Pharmacol. Sin..

[19] Yu T, Sun S (2023). Role and mechanism of ferroptosis in acute lung injury. Cell cycle.

[20] Xiang W, Yi X, Xue-Hai Z, Ding-Sheng J (2020). Posttranslational modifications in ferroptosis. Oxid. Med. Cell. Longev..

[21] Li J (2020). Ferroptosis: Past, present and future. Cell. Death Dis..

[22] Shi Z-Z (2021). Prognostic and immunological role of key genes of ferroptosis in pan-cancer. Front. Cell Dev. Biol..

[23] Lee JR (2017). Overexpression of glutathione peroxidase 1 predicts poor prognosis in oral squamous cell carcinoma. J. Cancer Res. Clin. Oncol..

[24] Fukuda M (2021). Down-regulation of glutathione peroxidase 4 in oral cancer inhibits tumor growth through SREBP1 signaling. Anticancer Res..

[25] Killion EA (2018). A role for long-chain acyl-CoA synthetase-4 (ACSL4) in diet-induced phospholipid remodeling and obesity-associated adipocyte dysfunction. Mol. Metab..

[26] Feng Y, Kang X, Li C, Nie M (2013). Expression of cytokeratin 19 and connexin 43 in 4-nitroquinoline-1-oxide-induced rat tongue carcinogenesis. Hua Xi Kou Qiang Yi Xue Za Zhi.

[27] Peng W, Li C, Ni H, Li H (2012). The expression of CK19 and anti-oncogene pien in oral squamous cell carcinoma. J. Luzhou Med. Coll..

[28] Zhong L-P (2006). Increased levels of CK19 mRNA in oral squamous cell carcinoma tissue detected by relative quantification with real-time polymerase chain reaction. Arch. Oral Biol..

[29] Malhotra R (2016). Correlation of Cyfra 21–1 levels in saliva and serum with CK19 mRNA expression in oral squamous cell carcinoma. Tumour Biol..

[30] Rajeswari P (2020). Expression of CK 19 as a biomarker in early detection of oral squamous cell carcinoma. J. Oral Maxillofac. Pathol..

[31] Wu F (2002). CYFRA 21–1 is released in TNF-alpha-induced apoptosis in the hepatocellular carcinoma cell line HuH-7. Int. J. Oncol..

[32] Wang Z (2023). FTO sensitizes oral squamous cell carcinoma to ferroptosis via suppressing ACSL3 and GPX4. Int. J. Mol. Sci..

[33] Hassannia B, Vandenabeele P, Berghe T (2019). Targeting ferroptosis to iron out cancer. Cancer Cell.

[34] Nakamura T, Naguro I, Ichijo H (2019). Iron homeostasis and iron-regulated ROS in cell death, senescence and human diseases. Biochim. Biophys. Acta Gen. Subj..

[35] Maiorino M, Conrad M, Ursini F (2018). GPx4, lipid peroxidation, and cell death: Discoveries, rediscoveries, and open issues. Antioxid. Redox Signal..

[36] Zhu T (2019). Ferroptosis promotes photodynamic therapy: Supramolecular photosensitizer-inducer nanodrug for enhanced cancer treatment. Theranostics.

[37] Wang Y (2022). Wnt/beta-catenin signaling confers ferroptosis resistance by targeting GPX4 in gastric cancer. Cell Death Differ..

[38] Lei G (2020). The role of ferroptosis in ionizing radiation-induced cell death and tumor suppression. Cell Res..

[39] Doll S (2017). ACSL4 dictates ferroptosis sensitivity by shaping cellular lipid composition. Nat. Chem. Biol..

[40] Yang Y (2022). ACSL3 and ACSL4, distinct roles in ferroptosis and cancers. Cancers (Basel).

[41] He Z (2021). Role of ferroptosis induced by a high concentration of calcium oxalate in the formation and development of urolithiasis. Int. J. Mol. Med..

[42] Huang J, Chen G, Wang J, Liu S, Su JJB (2022). Platycodin D regulates high glucose-induced ferroptosis of HK-2 cells through glutathione peroxidase 4 (GPX4). Bioengineered.

[43] Wanberg LJ (2021). Prevalence of sleepiness and associations with quality of life in patients with sleep apnea in an online cohort. J. Clin. Sleep Med. JCSM Off. Publ. Am. Acad. Sleep Med..

[44] Zhang X (2021). Endogenous glutamate determines ferroptosis sensitivity via ADCY10-dependent YAP suppression in lung adenocarcinoma. Theranostics.

