# Supplementary material for: Silencing CK19 regulates ferroptosis by affecting the expression of GPX4 and ACSL4 in oral squamous cell carcinoma in vivo and in vitro
Source: Sci Rep. 2024 Jul 10;14:15968. doi: 10.1038/s41598-024-65079-0 (PMC11237079; doi:10.1038/s41598-024-65079-0)
Supplement: Supplementary file 1 — Supplementary Figure 1. [file 41598_2024_65079_MOESM1_ESM.pdf]

Supplementary table 1 Interference sequence of lentivirus

genes	Interference sequences (5' to 3')
pHS-ASR-1115	CAGGAAGATCACTACAACAAT
pHS-ASR-1116	GCGCTGATCAGCGGTATTGAA
pHS-ASR-1117	AGGTCAGTGTGGAGGTGGATT
pHS-ASR-LW429	AAACGTGACACGTTCGGAGAA

Supplementary table 2 The primer sequences of CK19 and GAPDH

genes	primer sequences (5' to 3')	bp	Tm (℃)
CK19	ACGGCAGCTAGAGGTGAAGATC	85	61.85
	GGTCGTGTAGTAGTGGCTGTAGTC		62.21
GAPDH	GACAAGCTTCCCGTTCTCAG	106	58.57
	CAATGACCCCTTCATTGACC		56.38
