# Supplementary figures and images for: Silencing CK19 regulates ferroptosis by affecting the expression of GPX4 and ACSL4 in oral squamous cell carcinoma in vivo and in vitro
Source: Sci Rep. 2024 Jul 10;14:15968. doi: 10.1038/s41598-024-65079-0 (PMC11237079; doi:10.1038/s41598-024-65079-0)

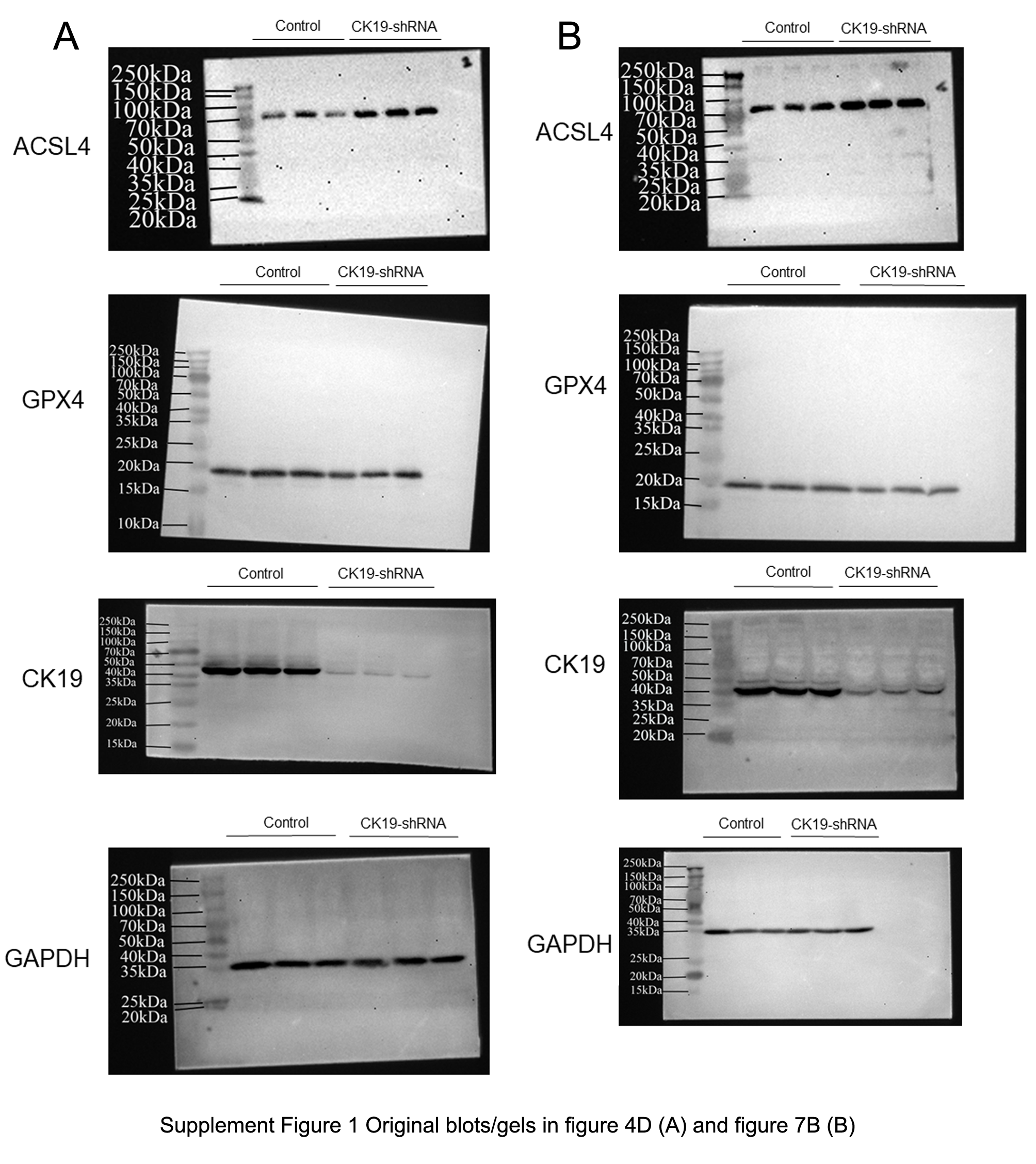

Supplement: Supplementary file 2 — Supplementary Tables. [file 41598_2024_65079_MOESM2_ESM.tif]
